# Alignment in spatial memory: Encoding of reference frames or of relations?

**DOI:** 10.3758/s13423-020-01791-y

**Published:** 2020-10-06

**Authors:** Holger Schultheis

**Affiliations:** Bremen Spatial Cognition Center, Institute for Artificial Intelligence, University of Bremen, Am Fallturm 1, 28359 Bremen, Germany

**Keywords:** Spatial memory, Reference frames, Judgment of relative directions, Inter-object relations

## Abstract

A common assumption about spatial memory is that it is organized along one or more reference directions such that access to memory is easier along directions aligned with the reference direction(s). This assumption rests to no small part on frequently replicated alignment effects arising in judgment of relative direction. In this contribution, we report an experiment designed to investigate a possible alternative explanation of alignment effects. By contrasting performance in a judgment of relative direction task with performance in an ego perspective taking task, we tested to what extent alignment effects arise from encoding of relations in addition to or instead of from organization along reference directions. Experimental results suggest little if any contribution of relation encoding on alignment effects, thus lending further support to the assumption of reference directions in spatial memory. Data from both tasks yielded the same alignment effects and provided evidence for a single direction being encoded in memory. Moreover, our results shed new light on and raise questions concerning differential sensorimotor and cognitive influence on spatial memory use. While both influence memory use, systematic bias seems to arise solely from reference directions, along which memory is organized.

**Electronic supplementary material:**

The online version of this article (10.3758/s13423-020-01791-y) contains supplementary material, which is available to authorized users.

Memory for spatial locations and layouts of objects in one’s surrounding is a fundamental aspect of human cognition. When setting the table, for example, spatial memory is essential to retrieve information of where required utensils (silverware, plates, etc.) can be found. Likewise, navigating to locations, which cannot be perceived directly, relies on memory of spatial layouts.

In line with its importance, considerable research has been devoted to examining the nature of spatial memory representation, organization, and access (Riecke & McNamara, 2017; Waller & Hodgson, 2006; Brandt et al., 2015; Chrastil & Warren, 2014; Hinterecker et al., 2019; Lavenex et al., 2015). A prominent assumption in previous research has been that enduring spatial memories are organized with respect to a reference frame (Meilinger, 2008; Mou et al., 2004; Street & Wang, 2016). Access to memory is assumed to be faster and less error-prone along certain directions, called *reference directions*, defined by the reference frame.

Evidence for such memory organization comes from empirical studies asking participants to perform *judgment of relative directions* (JRD). In a JRD task, participants first memorize a layout of objects surrounding them. Once the layout is sufficiently memorized, perceptual access to the layout is precluded and participants have to point to one of the objects as if standing at a second object and facing a third object of the layout. The direction from the second to the third object defines an imaginary facing heading. An often-replicated result of JRD studies are so-called *alignment effects* (Marchette et al., 2011; Shelton & McNamara, 2001; Meilinger & Bälthoff, 2013; Street & Wang, 2014; Kelly & McNamara, 2008), which are characterized by faster and less error-prone JRD performance for some imaginary headings than for others. Easier headings are usually headings that align with or are orthogonal to directions that are salient in the environment during learning such as, for example, the learners facing direction (Kelly et al., 2018) or directions aligned with axes of surrounding walls or axes of symmetry (Mou & McNamara, 2002).

The common interpretation of such alignment effects is that the directions that yield better JRD performance are directions along which enduring spatial memory, often assumed to store object-to-object relations (Sholl, 2001; Mou et al., 2004), is organized. However, another factor that may give rise to alignment effects has not been sufficiently investigated by previous research. In a JRD task, the imaginary heading is defined by the relation between two of the objects of the memorized layout. Therefore, determining which imaginary heading to adopt requires correctly retrieving the corresponding object-to-object relation from the enduring representation. If one is not able to remember the relation, the relation has to be inferred, which is arguably more time-consuming and error-prone. Consequently, alignment effects may arise because some object-to-object relations are more likely to be encoded than others.

Below we describe an experiment that has been designed to investigate this alternative explanation of alignment effects. An important assumption underlying the experiment is that the enduring spatial memory representation discussed above is complemented by a more transient representation, which has been called *sensorimotor representation* (SR). The SR encodes body-to-object relations to objects in the immediate surrounding. The encoded relations are thought to be easily and readily available as the representation constitutes the basis for motor actions in the immediate surrounding. Most current theories of spatial memory (Avraamides & Kelly, 2008; Wang, 2016; Byrne et al., 2007; Mou et al., 2004; Sholl, 2001; Waller & Hodgson, 2006; May, 2004) assume the existence of such a SR.

Against this background, the experiment compares performance in a JRD task with performance in an *ego perspective taking* (EPT) task. In EPT, participants learn an object layout as in a JRD task. After memorization, they are asked to point to one of the learned objects as if facing one of the other objects: The imaginary heading is defined by the relation between the participants’ body and one of the learned objects. Accordingly, the relation that defines the imaginary heading need not be retrieved from the enduring representation, but can be retrieved from the SR. As a result, in EPT, only adopting the imaginary heading and determining the correct pointing response, but not determining which imaginary heading to adopt requires access to the enduring representation. Consequently, the extent to which alignment effects in a JRD and an EPT task mirror each other provides evidence on how strongly the effect depends on either the need to determine the imaginary heading by retrieving object-to-object relations from the enduring representation or on the organization of the representation along reference directions. If alignment effects are similar for both tasks, alignment effects are unlikely to be driven by object-to-object relations. If, however, alignment effects are different for the two tasks, encoded relations may be at the core of the observed effects. The study of Mou et al., (2004) provided an initial hint that memory-based alignment effects may arise in EPT. Our study constitutes a more principled examination of the relative contribution of encoded relations and reference directions by (a) manipulating task within subject, (b) considering a wide range of imaginary headings, and (c) avoiding and removing possible influences of spatial updating and disparity effects (May, 2004). We also manipulated the shape of the object layout and the learning room to be either circular or square. In the square condition, based on previous research, we expected that the alignment effect takes the form of a *sawtooth pattern*, in which imaginary headings aligned with the learning heading or major room/layout axes are easier than headings misaligned with these directions. In the circular condition, the main axes are the body axes (front–back, left–right) and depending on how salient the axes are perceived to be by the participants we expected a similar sawtooth pattern to arise.

In addition to examining the presence of alignment effects in response time and absolute error, we also conducted a signed error analysis that has been shown to be indicative of possibly encoded reference directions (Street and Wang, 2014). Signed error is computed by subtracting the correct from the observed pointing direction. Signed error will be positive/negative, if the observed direction deviates counterclockwise/clockwise from the correct direction. Thus, a positive/negative average error for an imaginary heading indicates a systematic counterclockwise/clockwise pointing bias for responses in this heading. If, for example, average signed errors of -10^∘^, 0^∘^, and 10^∘^ are observed for imaginary headings 315^∘^, 0^∘^, and 45^∘^, respectively, this indicates that the responses in headings 315^∘^ and 45^∘^ are both biased towards 0^∘^. Such a bias pattern suggests that the central imaginary heading (called *attractor*, 0^∘^ in the example) is aligned with a reference direction encoded in memory. Consequently, attractors indicate reference directions encoded in memory. If alignment effects are found for imaginary headings that are not attractors, this has been argued to indicate ease of transformation rather than encoded reference directions see Street & Wang, (2014, for further detail).

## Methods

### Participants

Twenty-five students of the University of Bremen gave informed consent to participate in the experiment. They received either course credit or monetary compensation for participation. The ethics committee of the University of Bremen approved the experiment. One participant did not follow instructions and was excluded from analyses. Of the remaining 24 participants, 15 were female and nine were male. The number of participants was determined using G*Power 3 (Faul et al., 2007) assuming *α* = 0.05, a power of 1−β = 0.85, and a medium-to-strong effect size *f* = 1/3, because previous research has mostly reported strong alignment effects (e.g., Mou & McNamara, 2002; Street & Wang, 2014). Participants’ age ranged from 20 to 40 years with a mean age of 26.4 years.

### Materials and design

**Fig. 1:**
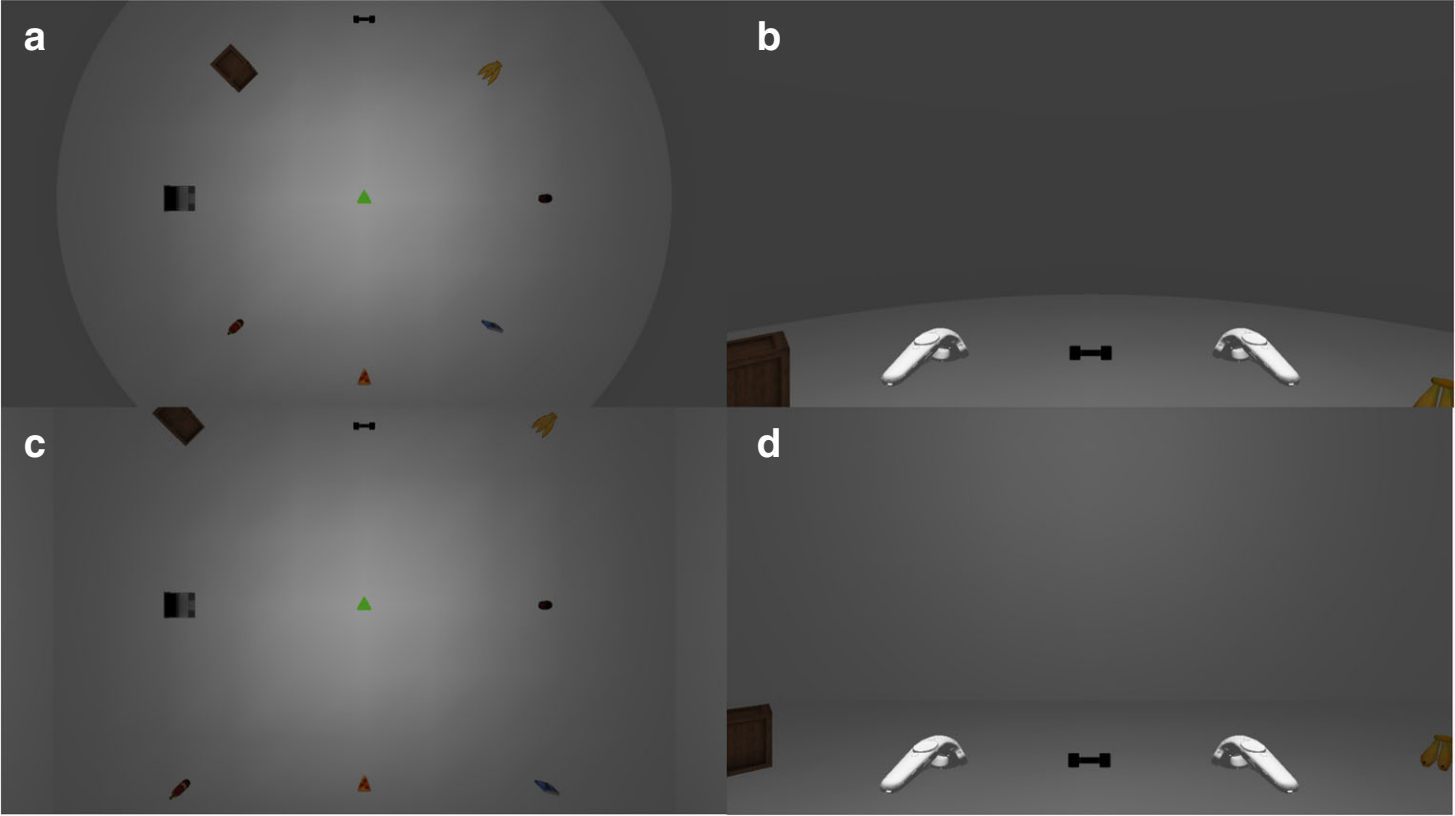
Bird’s eye and first-person view of the circular (**a**, **b**) and square (**c**, **d**) layouts used in the experiment

The complete experiment took place in virtual reality: All experimental instructions and material were presented through an HTC Vive Pro head-mounted display. Participants interacted with the experimental environment by two HTC Vive Controller (one held in each hand). The object layout consisted of eight objects (dumbbell, bananas, can, book, pizza slice, wine, laptop, crate) that were arranged either in a circle (Fig. 1a and b) or in a square (Fig. 1c and d) around the participant (indicated by the green triangle in the middle of Fig. 1a and c). The shape of the room in which the layout and the participant were located mirrored the shape of the object arrangement.

Experimental trials were constructed as follows. For the EPT task, imaginary headings were provided by naming an object, which the participant had to imagine to be facing. Consequently, each layout allowed eight unique headings that differed by 0^∘^, 45^∘^, 90^∘^, 135^∘^, 180^∘^, 225^∘^, 270^∘^, and 315^∘^ from the participant’s actual heading, respectively. For each of these headings, participants had to point to each of the seven objects, which did not define the imaginary heading. Accordingly, the EPT task consisted of 8 ∗ 7 = 56 trials resulting from the full combination of possible imaginary headings and remaining objects to point to.

For the JRD task, trials were selected to match the trials in the EPT task as closely as possible regarding the imaginary heading and the direction to which participants have to point within each imaginary heading. In the square layout, it was possible to match 52 of the 56 EPT trials. The layout does not allow a 180^∘^ pointing response for imaginary headings 45^∘^, 135^∘^, 225^∘^, and 315^∘^, because no matter how these imaginary headings are defined, there is no object behind the participant. Consequently, the JRD task in the square layout consisted of 52 trials. In the circular layout, only 32 JRD trials could be realized. To obtain more reliable measurements of performance within each imaginary heading, these 32 trials were presented twice yielding an overall of 64 trials for the JRD task in the circular condition. Participants first responded to the 32 trials in random order. Then the trials were presented again in random order subject to the constraint that a trial’s repetition occurred no earlier than ten trials after its first appearance.

Because previous research has shown that the disparity between the pointing direction from the imaginary heading and the pointing direction from the actual heading influences performance (May, 2004), JRD trials were selected to match EPT trials also in terms of disparity. Specifically, the average disparity across all JRD trials within an imaginary heading closely matched the average disparity across all EPT trials for the same imaginary heading. Average disparities in the EPT task were 0^∘^, 45^∘^, 90^∘^, 135^∘^, 180^∘^, 135^∘^, 90^∘^, 45^∘^ for imaginary headings 0^∘^ – 315^∘^, respectively. Average disparities in the JRD task were 22.5^∘^, 45^∘^, 90^∘^, 135^∘^, 157.5^∘^, 135^∘^, 90^∘^, 45^∘^ for imaginary headings 0^∘^ – 315^∘^, respectively.

Note that across all conditions, headings 0^∘^, 90^∘^, 180^∘^, and 270^∘^ were aligned with main body axes (front–back, left–right). For the square layout, these headings were also aligned with the main environmental axes. Headings 45^∘^, 135^∘^, 225^∘^, and 315^∘^ were misaligned with all main axes in all conditions.

Participants were randomly assigned to different object layout shapes. Consequently, the overall experimental design comprised the factors layout shape (between: circular, square), task (within: EPT, JRD), and imaginary heading (within: 0^∘^ – 315^∘^).

### Procedure

After putting on the head-mounted display and making themselves familiar with the controllers, participants were instructed to move into position (indicated by a green triangle on the floor of the virtual room, see Fig. 1).

The experiment started with a learning phase, in which participants had the opportunity to view the objects surrounding them. Participants were allowed to rotate their head and, insofar as necessary, their upper torso, but were not allowed to turn their whole body or to move their feet. After 30*s* the object layout disappeared and participants had to point to each of the objects. The object names were presented in random order on the head-mounted display. Participants were asked to point to the corresponding object with an extended arm such that the tip of the controller pointed towards the object. As during studying the layout, participants were allowed to rotate their head and upper torso but not allowed to move their feet during pointing. When participants pressed the track pad button on the controller, the pointing direction and the button-press time were recorded. If the absolute pointing error for one or more of the objects was larger than 45^∘^, the presentation of the object layout and the subsequent pointing test were repeated up to three times until the participant correctly pointed to all objects.

Once participants had sufficiently memorized the object layout, the second phase of the experiment began. In this phase, participants were not able to see the objects and performed a block of EPT trials and a block of JRD trials. The order of the tasks was counterbalanced across subjects. Before each block, participants were familiarized with the task by a corresponding practice trial involving prominent buildings on the university campus. Within each block the trials were shown one after the other in random order. EPT trials asked participants “Please point to *obj*_1_ as if facing *obj*_2_”, where *obj*_1_ and *obj*_2_ were names of different objects in the layout. JRD trials asked participants to “Please point to *obj*_1_ as if standing at *obj*_2_ and facing *obj*_3_”. After the end of the first block, participants were allowed to take a break before starting with the second block.

## Results

We analyzed response times, absolute pointing error, and signed pointing error. Response time was measured as the time from presentation of the problem statement until a track pad button was pressed. Absolute error was the absolute angular difference between the actual and the desired pointing direction. Signed error was computed by subtracting the desired from the actual pointing direction. The actual pointing direction was computed as the direction from the head-mounted display to the tip of the controller, whose track pad button was pressed. Standard deviations and means were computed for each individual and condition and values outside a 2 ∗ *SD* range from the mean were excluded from analyses (about 3*%* of all trials). An *α* of 0.05 was adopted as the level of significance for all statistical analyses.

### Response time and absolute error

Although participants were not able to see the objects during the second phase of the experiment, participants remained in the same (virtual) room as during the first phase. This has two consequences: First, in the 0^∘^ heading of the EPT task, people may rely on their SR to solve the task, which renders the interpretation of performance in the 0^∘^ heading problematic for the purposes of this study, which aims to investigate the properties of the enduring representation. Because performance in the 180^∘^ heading has been argued to be derived from the 0^∘^ heading (e.g., Rieser, 1989), the 0^∘^ and 180^∘^ headings were excluded when analyzing the EPT task^1^. Second, analyses have to take the disparity between pointing from the imaginary heading and pointing from the actual heading into account (May, 2004). Because the effect of disparity is not of main interest in this study, before further analyses, influence of disparity was partialed out as follows: for each participant, a linear regression with disparity as predictor was computed. Responses predicted by this model were subtracted from the observed values to obtain residuals. The participant average across all conditions was added to the residuals to avoid an artificial inflation of effect sizes. Data before partialing out disparity are given in Appendix A.

#### Response time

**Fig. 2:**
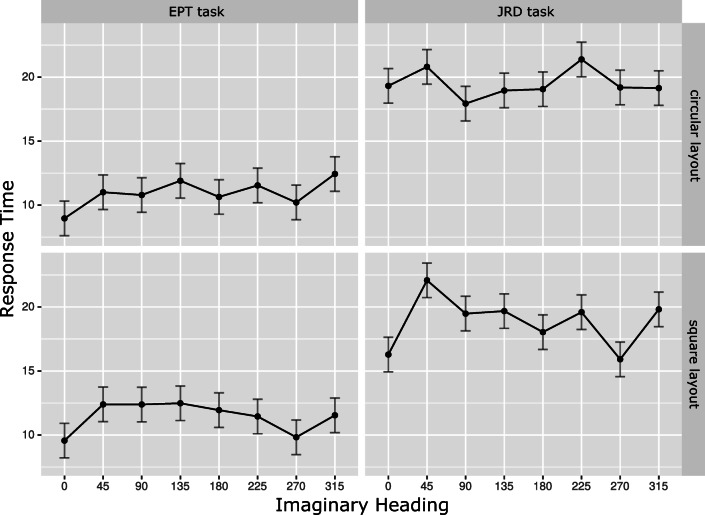
Response time (in s) for different imaginary headings. The top / bottom row shows times for the circular / square layout. The left / right column shows times for the EPT / JRD task. *Error bars* indicate the standard error

Figure 2 displays response times for each imaginary heading broken down by task and layout shape. The plots indicate two main effects in the data: First, participants took considerably longer to complete JRD tasks than to complete EPT tasks. Second, imaginary heading clearly influenced performance. Specifically, for both layouts and both tasks, the plots indicate a sawtooth pattern in which participants responded faster for imaginary headings aligned with main body and environmental axes than for misaligned imaginary headings. In particular, this pattern is also present for the EPT task.

Statistical analyses corroborated these impressions. A 2 (layout shape) x 2 (task) x 8 (imaginary heading) analysis of variance (ANOVA) revealed a main effect of task (*F*(1,22) = 41.44,*p* < 0.001,generalized *η*^2^ = 0.24) with responses in the EPT task (*M* = 11.32*s*,*SD* = 3.46*s*) being faster than responses in the JRD task (*M* = 19.17*s*,*SD* = 8.24*s*). The main effect of imaginary heading was also significant (*F*(3.88,85.32) = 3.39,*p* = 0.013,generalized *η*^2^ = 0.02). No other main effect or interaction reached significance (all *ps* > 0.35).

To more specifically check for the presence of a sawtooth pattern, we ran a second ANOVA with the factors layout shape, task, and alignment (aligned vs. misaligned). Headings 45^∘^, 135^∘^, 225^∘^, and 315^∘^ were considered misaligned. Headings 0^∘^, 90^∘^, 180^∘^, and 270^∘^ were considered aligned for the JRD task. For reasons mentioned above, 0^∘^ and 180^∘^ were not considered in the alignment analysis for the EPT task. We first checked for the presence of a sawtooth pattern across tasks and then, specifically, for the presence of a sawtooth pattern in the EPT task. Response times for aligned headings were significantly faster than response times in misaligned headings across tasks (*M* = 14.48*s*,*SD* = 5.16*s* for aligned and *M* = 16.01*s*,*SD* = 6.22*s* for misaligned; *F*(1,22) = 6.5,*p* = 0.018,generalized *η*^2^ = 0.014). The same pattern was present in the EPT task (*M* = 10.8*s*,*SD* = 3.73*s* for aligned and *M* = 11.84*s*,*SD* = 3.63*s* for misaligned) and reached marginal significance (*F*(1,22) = 3.98,*p* = 0.059,generalized *η*^2^ = 0.021). Except for the main effect of task (*F*(1,22) = 37.93,*p* < 0.001,generalized *η*^2^ = 0.27) no other main effects or interactions approached significance (all *ps* > 0.2).

#### Absolute error

**Fig. 3:**
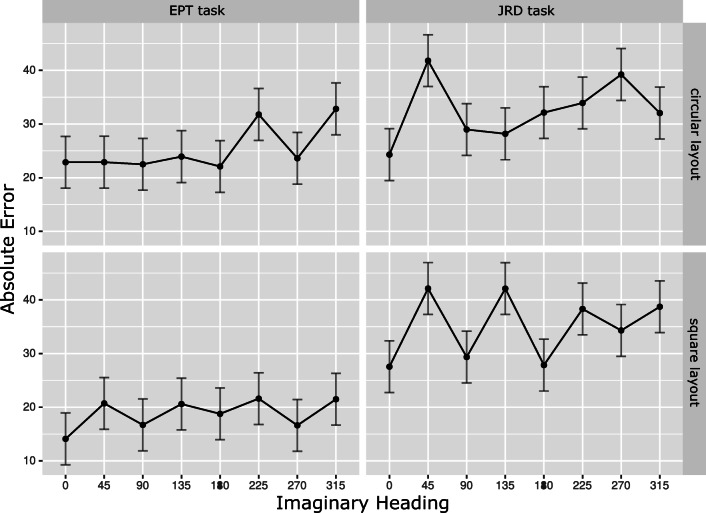
Absolute error (in degree) for different imaginary headings. The top / bottom row shows errors for the circular / square layout. The left / right column shows errors for the EPT / JRD task. *Error bars* indicate the standard error

Figure 3 displays absolute errors for each imaginary heading broken down by task and layout shape. As can be seen from the figure, absolute errors exhibit a similar pattern as response times. This is also mirrored in the results of the statistical analyses. The ANOVA revealed a significant effect of task (*F*(1,22) = 14.49,*p* < 0.001,generalized *η*^2^ = 0.1) with lower angular error in the EPT (*M* = 22.17^∘^,*SD* = 13.83^∘^) than in the JRD (*M* = 33.8^∘^,*SD* = 14^∘^) task. The main effect of imaginary heading was also significant (*F*(3.97,87.32) = 4.42,*p* = 0.003,generalized *η*^2^ = 0.04), while no other main effects or interactions reached significance (all *ps* > 0.15).

As for response times, a second ANOVA was conducted to more specifically check for alignment effects. The analysis indicates lower error for aligned than for misaligned headings across tasks (*M* = 25.16^∘^,*SD* = 13.29^∘^ for aligned and *M* = 30.81^∘^,*SD* = 10.58^∘^ for misaligned; *F*(1,22) = 16.33,*p* < 0.001,generalized *η*^2^ = 0.04). The same pattern was present in the EPT task (*M* = 19.86^∘^,*SD* = 16.69^∘^ for aligned and *M* = 24.47^∘^,*SD* = 12.69^∘^ for misaligned) and reached marginal significance (*F*(1,22) = 4.27,*p* = 0.051,generalized *η*^2^ = 0.026). Except for the main effect of task (*F*(1,22) = 13.87,*p* = 0.0012,generalized *η*^2^ = 0.14) no other main effects or interactions approached significance (all *ps* > 0.2).

### Signed error

Signed error analyses were conducted to investigate the possible presence of attractor effects as those reported in Street and Wang (2014): If the signed error increases from negative to positive across headings from 45^∘^ counterclockwise to 45^∘^ clockwise around a certain heading *H*, *H* is assumed to be an attractor (see, Street and Wang (2014), for detail). The attraction analyses allowed to examine to what extent our experiment replicated the results of Street and Wang (2014) and, in particular, to what extent the same attractors existed in the EPT and the JRD task. Because signed errors constitute circular data (an angle of -179^∘^ is very close to an angle of 179^∘^), we employed circular statistics (Jammalamadaka & SenGupta, 2001) for analyses.

**Fig. 4:**
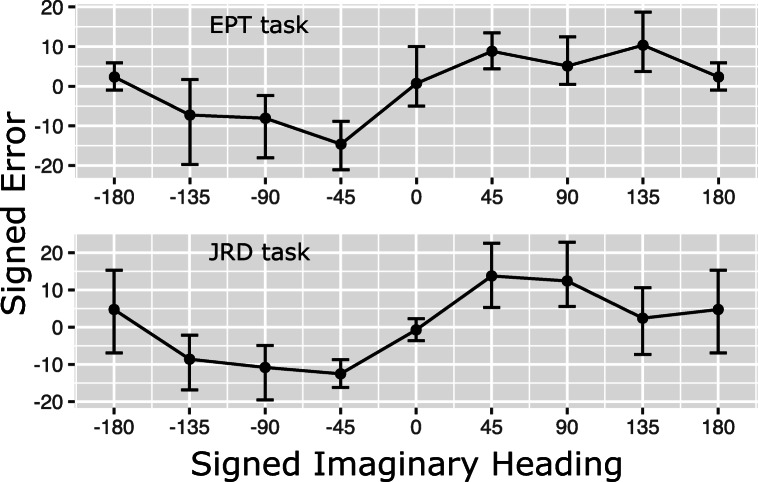
Signed errors (in degrees) for different imaginary headings. The *top panel* shows errors for the EPT task, the *bottom panel* shows errors for the JRD task. *Bars* indicate 95*%* confidence intervals

Average signed errors across imaginary headings are displayed in Fig. 4. As can be seen, signed errors were quite similar for the two tasks yielding a substantial and significant correlation (*r*(8) = .88,*p* = 0.029). More importantly, for both tasks, the plots indicate the 0^∘^ heading as an attractor: there is a clear rise in signed errors from the -45^∘^ heading (signed error around -13^∘^) over the 0^∘^ heading (error around 0^∘^) to the 45^∘^ heading (error around 10^∘^). None of the other headings shows a comparable pattern.

To further investigate attractor patterns, we conducted the following correlation analysis for all aligned headings *H*: We correlated headings *H*-45^∘^, *H*, *H*+ 45^∘^ with the corresponding signed errors for each participant and task. Then we determined the median correlation across participants for each task and used a sign test to determine whether the median correlation was significantly different from zero. The results of these analyses are provided in Table 1: The 0^∘^ heading is the only heading that exhibits a significant (and large) positive correlation, suggesting that the 0^∘^ heading is the only attractor for both the EPT and the JRD task.

**Table 1:** Median correlations of imaginary heading with signed error around aligned headings (see text for detail)

	0^∘^	90^∘^	180^∘^	-90^∘^
EPT task	0.91*	0.02	− 0.53	− 0.55
JRD task	0.95*	− 0.71	− 0.19	− 0.45

Asterisks indicate significance

### Discussion

Our results provide preliminary evidence that the observed alignment effects arise mainly from reference directions encoded in memory and only to little (if any) extent from differential encoding of inter-object relations^2^. First, the fact that EPT was significantly faster and less error-prone than JRD supports our initial assumption that people rely on their SR for determining imaginary headings in EPT. Second, for both response time and absolute error, behavior in EPT exhibited a sawtooth pattern that was not significantly different from the pattern observed in the JRD task. Third, the pattern of signed errors across imaginary headings was very similar for the EPT and the JRD task and, in particular, in both tasks the 0^∘^ imaginary heading was the only attractor heading. If, as previously argued (Street & Wang, 2014), the attractor at 0^∘^ indicates that spatial memory is organized along a single reference direction, our results imply that (a) performance in both tasks relies on similar memory organization and (b) this memory is organized along a single predominant reference direction. As such, our findings also suggest that the alignment effect observed for headings 90^∘^, 180^∘^, and 270^∘^ is due to easier transformations and not due to a representation of these headings in memory. It appears that while only the learning heading was encoded in memory, other salient axes in the environment (e.g., intrinsic layout axes) influenced the ease with which participants were able to transform their actual heading to corresponding imaginary headings.

Because the participants’ body remained in the learning position and orientation (0^∘^) throughout the experiment, one may wonder to what extent the observed alignment and attractor effects arose from an influence of the SR on adopting imaginary headings^3^. This is an intriguing idea, in particular also because none of the existing major theories of spatial memory (Avraamides & Kelly, 2008; Wang, 2016; Mou et al., 2004; Sholl, 2001; Waller & Hodgson, 2006) explicate such a role of the SR. On the other hand, it seems unlikely that the SR should be the sole cause for the observed effects. Several previous studies, ruling out an influence of the SR, have reported the same alignment (e.g., Shelton & McNamara, 2001) and attractor (Street & Wang, 2014) effects. If there would be any influence of the SR, it seems reasonable to suppose that its effects would combine with the previously established effects arising from the enduring representation. Specifically, any effect arising from the enduring representation would still be present in the participants’ response and one would still expect to see a difference between EPT and JRD tasks, if alignment were due to encoded relations.

Furthermore, the alignment and attractor effects we found in our experiment indicate no additional influence of the SR. In particular, the pointing bias as exhibited by the attractor effect is of the same size as the one reported by Street and Wang (2014). This is remarkable, because previous studies have provided evidence for the impact that (knowledge of) one’s own body’s pose has on performance in both JRD (Mou et al., 2004) and EPT (May, 1996; Kelly et al., 2007) tasks. One possible explanation is that the actual body pose interferes with pointing from imaginary headings, but does not systematically distort pointing responses to a particular direction. This is in line with the signed error analyses reported by May (2004), which revealed pointing responses pulled towards the body pose as well as pointing responses repelled by the body pose. The direction along which memory is organized, in contrast, seems to exhibit a notable pull effect on participants’ pointing responses (Street and Wang, 2016). Jointly with the existing findings, our results suggest that although body pose influences performance this influence is undirected while pointing bias seems to arise mainly from memory organization.

Further corroboration and investigation of these findings and implications is desirable, partly because alignment effects in the EPT task closely missed significance and partly because current theorizing remains underspecified regarding key properties of representations and processes.

## Conclusions

It appears that differential encoding of relations plays a minor (if any) role in alignment effects observed in experiments employing judgment of relative directions. This lends additional support to the importance of encoded reference directions for spatial memory access. It also suggests that which relations are encoded and maintained in memory is not systematically influenced by axes that are salient during the learning of the spatial layout. Furthermore, sensorimotor interference by one’s body pose does not seem to contribute to pointing bias as arising from encoded reference directions. This suggests that bias is a mainly cognitive phenomenon and, thus, that cognitive and sensorimotor influences on adopting imaginary headings are moderated by distinct underlying mechanisms. The nature of these mechanisms and their interplay remains to be elucidated. While most existing theories are consistent with the idea of separate cognitive and sensorimotor influences, they do not provide sufficient mechanistic detail to offer an explanation for the fact that bias should arise from one but not the other influence. Future work is required to examine in more detail the mechanisms and representation structures that underlie spatial memory encoding, maintenance, and access.

## Electronic supplementary material

Below is the link to the electronic supplementary material.

(PDF 1.28 MB)

## Figures and Tables

**Fig. 5:**
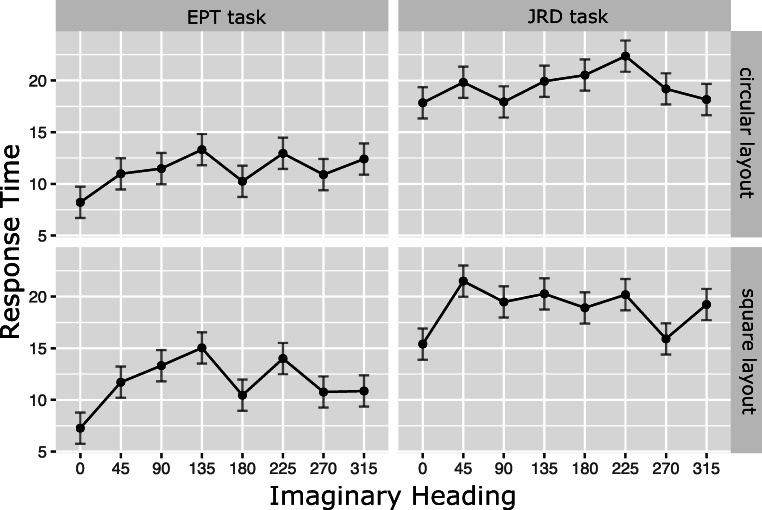
Response time (in s) for different imaginary headings. The top / bottom row shows times for the circular / square layout. The left / right column shows times for the EPT / JRD task. *Error bars* indicate the standard error

**Fig. 6:**
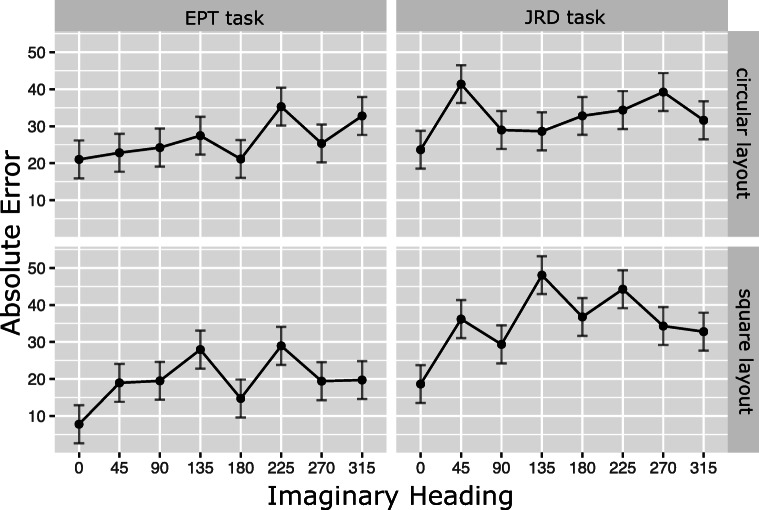
Absolute error (in degrees) for different imaginary headings. The top / bottom row shows errors for the circular / square layout. The left / right column shows errors for the EPT / JRD task. *Error bars* indicate the standard error

## Appendix A: Raw Response Time and Absolute Error

Response time and absolute error with disparity effects are displayed in Figs. 5 and 6, respectively.

## Footnotes

1 Effects were the same, though partly stronger, when 0^∘^ and 180^∘^ were included in the analyses.

2 To investigate to what extent our results are influenced by asking participants to work on the two perspective taking tasks consecutively, we conducted the same analyses using only the data of each participant’s first task. For response times and signed errors, the effects and effect sizes remained virtually unchanged. For absolute errors, the effects were the same, but effect sizes tended to be smaller. While the reduced effect sizes in absolute error suggest a perspective taking familiarization effect across tasks (supported by notably increased standard deviations), the analyses generally indicate that the within-subject design did not have any substantial impact on the found effects.

3 We thank an anonymous reviewer for suggesting this.
